# Lifetime cost-effectiveness of lecanemab for early Alzheimer’s disease

**DOI:** 10.3389/fpubh.2026.1692508

**Published:** 2026-01-30

**Authors:** Xiangxiang Jiang, Gang Lv, Morgan E. Mendes, Jun Wu, Jing Yuan, Z. Kevin Lu

**Affiliations:** 1 Department of Clinical Pharmacy and Outcomes Sciences, College of Pharmacy, University of South Carolina, Columbia, SC, United States; 2 Department of General Surgery, The First Medical Center of Chinese PLA General Hospital, Beijing, China; 3 Department of Sociobehavioral and Administrative Pharmacy, Nova Southeastern University, Fort Lauderdale, FL, United States; 4 Institute of Chinese Medical Sciences, University of Macau, Taipa, Macao SAR, China

**Keywords:** aducanumab, Alzheimer’s disease, cost-effectiveness analysis, lecanemab, Markov model

## Abstract

**Introduction:**

Lecanemab is the second anti-amyloid-*β* monoclonal antibody to receive FDA approval for the treatment of early Alzheimer’s disease (AD), following aducanumab. Unlike aducanumab, which faced restricted Medicare coverage, lecanemab received traditional approval in 2023, resulting in broader access through Medicare. Despite these developments, the comparative cost-effectiveness of lecanemab and aducanumab has not been fully established. This study aimed to assess whether lecanemab is more cost-effective than aducanumab in the management of early AD.

**Methods:**

An indirect comparison of cost-effectiveness was performed due to the absence of head-to-head randomized controlled trials. A five-state Markov model was constructed from the perspective of the US healthcare system with a lifetime horizon and a 1-year cycle length. Model outcomes included life-years (LYs), quality-adjusted life-years (QALYs), and costs, all discounted at an annual rate of 3%. Incremental cost-effectiveness ratios (ICERs) were calculated and compared with established willingness-to-pay (WTP) thresholds. One-way sensitivity analyses identified key drivers of model uncertainty, and a probabilistic sensitivity analysis (PSA) with 1,000 Monte Carlo simulations tested the robustness of the findings.

**Results:**

The incremental cost of the lecanemab group compared to the aducanumab group was $30,018.97, with an increase in quality-adjusted life-years (QALYs) of 0.25, resulting in an ICER of $121,678.49 per QALY gained. The result of the one-way sensitivity analysis showed that the utility of the state of mild dementia due to AD had the most important effects on the ICER of the lecanemab group compared to the aducanumab group. The probabilistic sensitivity analysis showed that lecanemab was more cost-effective than aducanumab across various WTP thresholds.

**Conclusion:**

Our findings suggest that lecanemab provides greater value than aducanumab; however, at current list prices, neither drug is cost-effective compared with the standard of care. Price reductions are necessary to improve affordability, particularly for lecanemab, which is more widely covered by Medicare. Policy implications remain significant, as under the Inflation Reduction Act (IRA), biologics such as lecanemab are exempt from Medicare price negotiations for 13 years post-approval, limiting short-term opportunities for cost adjustment.

## Introduction

Alzheimer’s disease (AD) is a brain disorder in which neurons are progressively damaged, leading first to memory, language, and thinking problems, and later to mood, personality, and behavioral changes (1). In 2025, an estimated 7.2 million Americans aged 65 and older will be living with Alzheimer’s dementia (1). By 2050, this number is projected to nearly double, reaching close to 13 million (1). The economic burden is also immense, with health and long-term care costs expected to rise from $384 billion in 2025 to approximately $1 trillion by 2050 (1). In 2024, unpaid caregivers contributed over 19 billion hours of care, valued at more than $413 billion (1).

Traditional treatments for AD have primarily focused on managing or alleviating symptoms linked to cognitive decline (2). These drugs reversibly inhibit the enzyme that breaks down acetylcholine, thus increasing the amount of acetylcholine available for synaptic transmission in the central nervous system (CNS), leading to better communication between neurons (3–5). However, new therapies for AD are designed to slow cognitive and functional decline by targeting the underlying disease process, specifically the accumulation of amyloid-*β* (Aβ) peptides in the brain, which form plaques that disrupt neuronal communication and trigger neurodegeneration.

Notably, aducanumab and lecanemab are the first two approved drugs of Aβ-targeted therapies. Two global, phase III, randomized, double-blind, placebo-controlled clinical trials (EMERGE and ENGAGE) were conducted to evaluate the efficacy of aducanumab (6). The EMERGE trial met its primary endpoint, showing a 22% reduction in clinical decline on the Clinical Dementia Rating–Sum of Boxes (CDR-SB) with high-dose aducanumab, while the ENGAGE trial did not meet its primary or secondary endpoints, contributing to controversial and inconsistent overall results (6). Regardless, the US Food and Drug Administration (FDA) approved aducanumab in June of 2021 for the treatment of AD in patients with mild cognitive impairment (MCI) and confirmed A*β* pathology before treatment initiation (7). Lecanemab became the second anti-amyloid-β (Aβ) therapy to gain FDA approval, receiving accelerated approval in January 2023 and later full approval in July 2023 (8), after the prior accelerated approval of aducanumab. In the phase III trial, lecanemab slowed clinical decline by 27% on CDR-SB over 18 months and improved key secondary measures, while also reducing amyloid plaques and phosphorylated tau levels (9, 10). The positive effects of lecanemab are thought to stem from its binding profile, as it preferentially targets soluble Aβ protofibrils, whereas aducanumab and several other monoclonal antibodies primarily bind to more aggregated forms of Aβ.

In April 2022, the Centers for Medicare and Medicaid Services (CMS) issued a national policy granting coverage for aducanumab only to patients enrolled in approved clinical studies (11). After lecanemab received full FDA approval in July 2023, CMS announced it would broaden access, allowing Medicare coverage for lecanemab when prescribed according to the FDA label and with participation in a qualifying registry to collect real-world data (12).

Although lecanemab appears to offer greater clinical benefits than aducanumab, it may also entail substantially higher costs, including administration expenses. Previous studies found that threshold prices for aducanumab vary widely, ranging from $3,000 to $22,800 (13–15). The estimated threshold price for lecanemab, however, falls in the lower end of this range (16), suggesting that lecanemab may need more reduction in drug price. As aducanumab is set to be discontinued by its manufacturer (Biogen) in 2024 to promote lecanemab (17), additional pieces of evidence are required to justify this decision and those made by the CMS. Therefore, this study aimed to evaluate the cost-effectiveness of lecanemab vs. aducanumab using an indirect comparison with the Markov model.

## Materials and methods

### Study design

Indirect comparisons of cost-effectiveness analyses were used to determine incremental costs and effects between lecanemab and aducanumab to account for the fact that no direct comparisons are available from randomized controlled clinical trials. A five-state Markov model was constructed to evaluate the cost-effectiveness of using lecanemab along with the standard of care (SoC) and aducanumab along with SoC compared to using SoC alone, from the perspective of the healthcare system over a lifetime horizon. The model was broken down to a 1-year cycle length to estimate the cost-effectiveness of lecanemab over patients’ entire lifetime. It considered outcomes such as life years (LYs), quality-adjusted life-years (QALYs), and costs, all of which were discounted at a rate of 3% annually in accordance with the recommendations of the Second Panel on Cost-Effectiveness in Health and Medicine (18). Incremental cost-effectiveness ratios (ICERs) were calculated and compared with the willingness-to-pay (WTP) threshold to determine cost-effectiveness.

**Figure 1:**
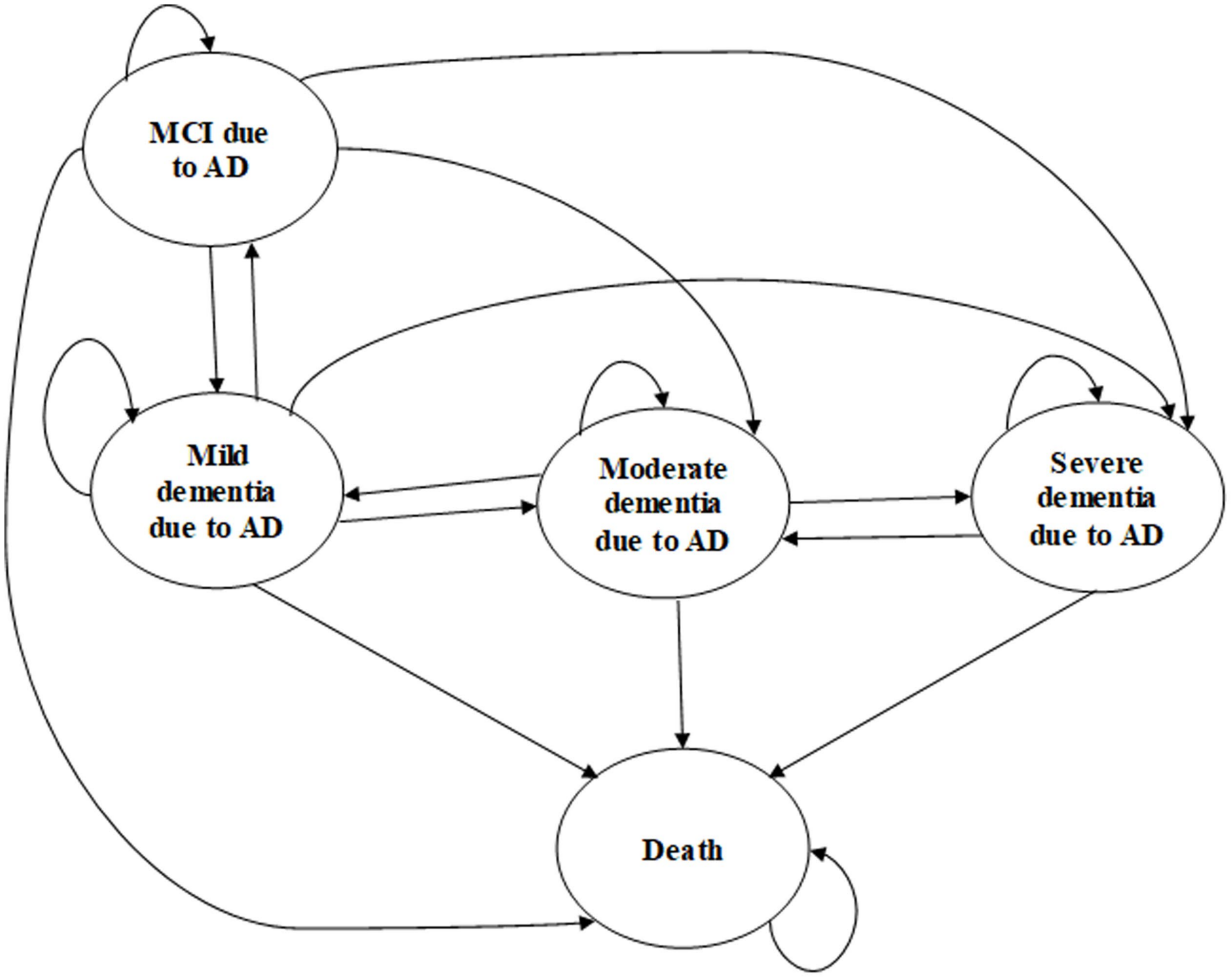
Markov model illustrating disease progression in Alzheimer’s disease. MCI, mild cognitive impairment; AD, Alzheimer’s disease.

The study group consisted of patients taking lecanemab or aducanumab with SoC, while the control group only used SoC. The five health states in the model included MCI due to AD, mild dementia due to AD, moderate dementia due to AD, severe dementia due to AD, and death (Figure 1). Annual transition probabilities between the health states of the control group were obtained from a recent analysis of AD progression (19), and we calculated transition probabilities of the study group using a hazard ratio (HR) obtained from randomized controlled trials (RCTs).

The starting population in the model was those with early AD (MCI or mild dementia due to AD). The distribution of the simulating cohort in the starting states of the model was based on a phase 3 RCT of lecanemab (10). In this RCT, 61.5% of patients in the lecanemab and 66.2% of patients in the SoC group started in the MCI health state, while the rest started in the mild dementia due to AD health state. The average age in the lecanemab group and SoC group was 71.4 and 71.0, respectively. To compare the lecanemab and aducanumab groups, we assumed that the basic population information in the aducanumab group was the same as in the lecanemab group. The model was implemented using Microsoft Excel.

### Model inputs

The primary clinical inputs in the model were the transition probabilities among alive health states, mortality rates, the rate of occurrence of amyloid-related imaging abnormalities (ARIAs), utilities, and costs (Table 1).

**Table 1:** Model parameters.

Parameters	Base case mean value	SE	Lower limit in OSA	Upper limit in OSA
Initial age of the lecanemab group	71.40	0.27	65.00	75.00
Initial age of SoC group	71.00	0.26	65.00	75.00
HR of lecanemab to SoC				
MCI to mild	0.69	NA	0.55	0.83
Mild to moderate	0.69	NA	0.55	0.83
Transition probability, SoC, 1-year				
MCI to mild	0.32	NA	0.26	0.38
MCI to moderate	0.04	NA	0.03	0.05
MCI to severe	0.01	NA	0.01	0.01
Mild to MCI	0.03	NA	0.02	0.04
Mild to moderate	0.36	NA	0.29	0.43
Mild to severe	0.05	NA	0.04	0.06
Moderate to MCI	0.00	NA	0.00	0.00
Moderate to Mild	0.04	NA	0.03	0.05
Moderate to severe	0.40	NA	0.32	0.48
Severe to MCI	0.00	NA	0.00	0.00
Severe to mild	0.00	NA	0.00	0.00
Severe to moderate	0.02	NA	0.02	0.02
Probability of ARIA occurrence, 1-year				
Lecanemab	0.15	NA	0.12	0.18
SoC	0.06	NA	0.05	0.07
Cost (US$)				
Lecanemab, per unit	26,500.00	NA	21,200.00	31,800.00
Administration	3,654.17	NA	2,923.34	4,385.01
Outpatient	3,237.26	251.30	2,589.81	3,884.72
Inpatient	17,935.84	1,145.99	14,348.67	21,523.01
ARIA of the lecanemab group	154.82	NA	123.86	185.78
ARIA of the SoC group	66.73	NA	53.39	80.08
Utility				
MCI	0.73	0.02	0.58	0.88
Mild	0.69	0.01	0.55	0.83
Moderate	0.53	0.01	0.42	0.64
Severe	0.38	0.01	0.30	0.46
Mortality, 1-year				
65–74	0.02	NA	0.02	0.03
75–84	0.05	NA	0.04	0.06
≥85	0.16	NA	0.13	0.19
HR for mortality at dementia stages				
MCI	1.82	0.16	1.46	2.18
Mild	2.92	0.19	2.34	3.50
Moderate	3.85	0.27	3.08	4.62
Severe	9.52	0.37	7.62	11.42
Discount rate				
Lecanemab group	0.03	NA	0.00	0.08
SoC group	0.03	NA	0.00	0.08

SE, standard error; OSA, one-way sensitivity analysis; HR, hazard ratio; SoC, standard of care; MCI, mild cognitive impairment; AD, Alzheimer’s disease; ARIA, amyloid-related imaging abnormalities.

The transition probabilities for the control group were obtained from a previous analysis of AD progression (19). The HR for progressing from MCI due to AD and from mild dementia due to AD to moderate dementia due to AD for the lecanemab group compared to the SoC group was 0.69, which came from the phase 3 RCT of lecanemab (10). The HR of MCI due to AD to mild dementia due to AD for the aducanumab group compared to the SoC group was 0.83, which was obtained from combining two phase 3 RCTs of aducanumab (6). The model assumed that the HRs for other transitions between the lecanemab and the SoC group, as well as the aducanumab group and the SoC group, were equal to 1.

The HRs of alive health states to death were applied to the age-adjusted all-cause mortality rate to determine the death transition probabilities of each health state. Specifically, the HRs were obtained from a longitudinal cohort study of 3,346 individuals aged 65–84 years, followed for 14 years (20), while the age-adjusted all-cause mortality rate came from the published data of the Centers for Disease Control and Prevention (CDC) (21). Information on the ARIA occurrence was sourced directly from the RCT of lecanemab and aducanumab (6, 10). The utilities of each health state were taken from a previous study (22).

The total costs in the model included the expense of lecanemab or aducanumab medication, administration costs, outpatient costs, inpatient costs, and ARIA costs. As stated in the report, the annual cost of lecanemab was $26,500 (23). The first-year cost of aducanumab was reported to be $20,500, while in the subsequent year, it was $28,200 (24). The drugs were given intravenously, and the administration costs were obtained from the Centers for Medicare and Medicaid Services (CMS) Physician Fee Schedule (25). The outpatient and inpatient costs of AD patients were obtained using claims data from the Medicare Current Beneficiary Survey (MCBS) (26). MCBS is a nationally comprehensive and authoritative survey linked with claims data of Medicare beneficiaries, which is sponsored by the CMS. AD-specific costs were calculated using the ICD-10 codes of AD (G30). The consumer price index (CPI) was used to adjust the outpatient and inpatient costs for the study year (27). In this model, only ARIA was considered to require treatment. If an ARIA event occurs, patients typically receive brain Magnetic Resonance Imaging (MRI) monitoring at 4-week intervals until the event has resolved or stabilized (28). Because the average ARIA duration lasts approximately 12 weeks, management of a single ARIA event generally requires three additional MRI scans (28). The unit price for MRI was obtained from the CMS Physician Fee Schedule (25). Therefore, the annual ARIA-related costs for each group were calculated as: 3 × unit MRI cost × annual ARIA rate.

### Base case analysis

In the base case analysis, incremental analysis was performed to determine cost-effectiveness. The study group was deemed cost-effective if the ICER was lower than the WTP threshold of $150,000 per QALY gained in the US (29). Otherwise, it would not be cost-effective.

### Sensitivity analyses

We conducted the one-way sensitivity analysis (OSA) to assess the uncertainty and robustness of the model. We used ±20% variation from the deterministic value to evaluate changes in the ICERs when a single input was changed, while the discount rate was between 0 and 8%. The results were summarized using a tornado diagram.

The probabilistic sensitivity analysis was also performed based on 1,000 Monte Carlo simulations to assess the effect of changes in multiple variables on the ICERs. In the probabilistic sensitivity analysis, random values were selected from the distribution of uncertain parameters, with the HRs following a log-normal distribution, utilities following a beta distribution, and costs following a gamma distribution. The mean of each parameter was consistent with the deterministic values, while standard error (SE) was used to determine the upper and lower limits of the distribution. If the SE was not reported in the source, it was assumed to be one-tenth of the mean. The price of lecanemab or aducanumab did not change in the probabilistic sensitivity analysis, as it was fixed. In addition, the mortality rates did not vary because they were based on the US population.

In addition, we conducted sensitivity analyses using updated stage-specific health utility values extracted from the systematic review by Landeiro et al. (30). Detailed results are presented in Supplementary Table 1. For transitions without published HRs, we also conducted scenario analyses on varying HRs for unobserved effects on progression. Two scenarios were evaluated: a favorable scenario (HR = 0.8) and an unfavorable scenario (HR = 1.2). The results of these analyses are reported in Supplementary Table 2.

## Results

### Base case analysis

In the base case analysis (Table 2), the cost of the aducanumab group increased by $101,770.83 compared to the SoC group, with LYs and QALYs increasing by 0.08 and 0.08, respectively. Compared with the SoC group, the cost of the lecanemab group increased by $131,789.80, with LYs and QALYs increasing by 0.33 and 0.32, respectively. The ICERs of the aducanumab group and the lecanemab group compared to the SoC group were $1,354,544.47 per QALY gained and $409,488.34 per QALY gained, respectively.

**Table 2:** Base case analysis results.

Groups	Incremental costs, US$	Incremental LYs	Incremental QALYs	ICER, US$/LY	ICER, US$/QALY
Aducanumab vs. SoC	101,770.83	0.08	0.08	1,237,130.61	1,354,544.47
Lecanemab vs. SoC	131,789.80	0.33	0.32	395,747.84	409,488.34
Lecanemab vs. aducanumab	30,018.97	0.25	0.25	119,716.27	121,678.49

SoC, standard of care; LY, life year; QALY, quality-adjusted life year; ICER, incremental cost-effectiveness ratio.

In addition, the incremental cost of the lecanemab group compared to the aducanumab group was $30,018.97, with an increase in LYs of 0.25 and an increase in QALYs of 0.25, resulting in an ICER of $121,678.49 per QALY gained.

### Sensitivity analyses

**Figure 2:**
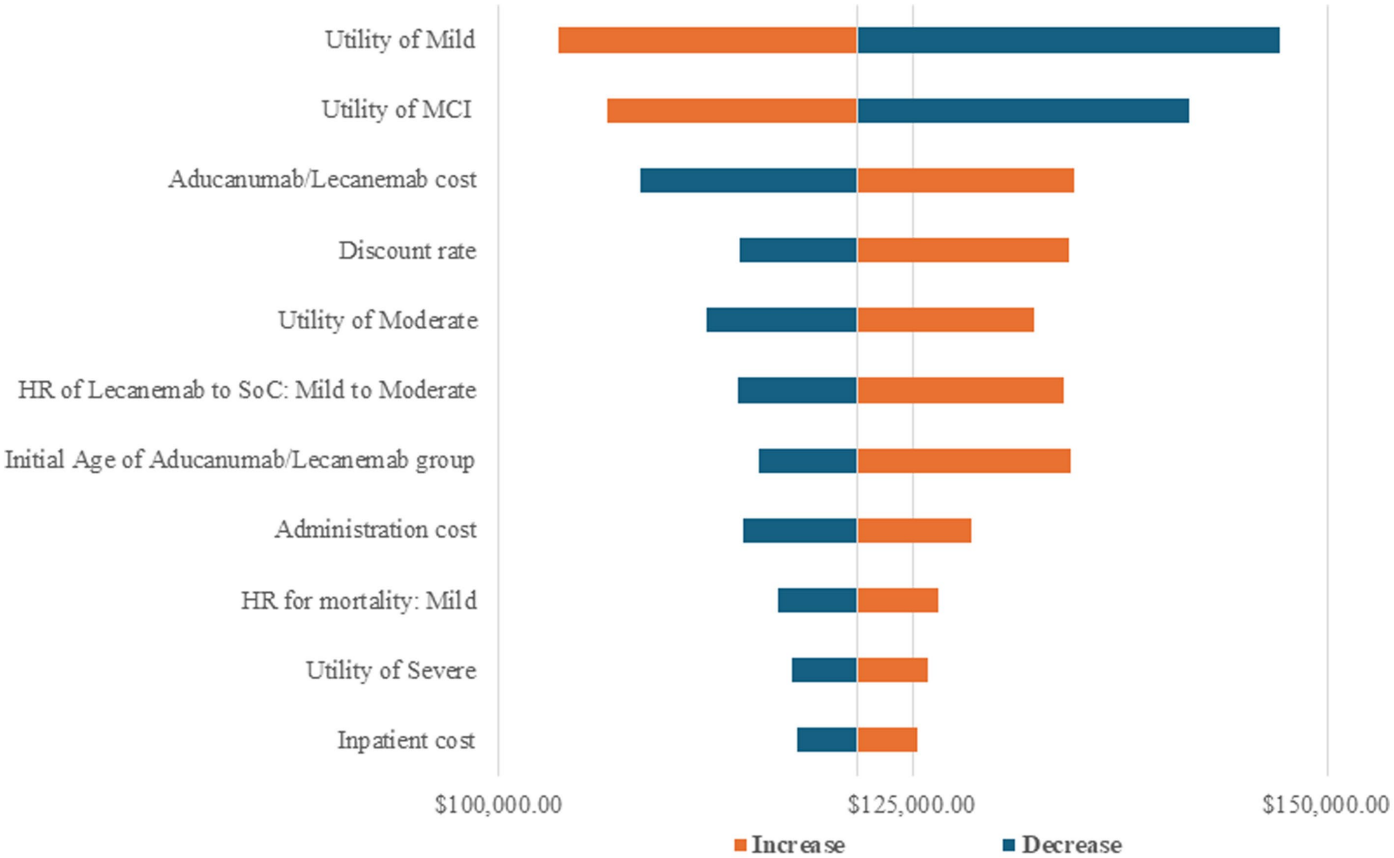
Tornado diagram of the result of one-way sensitivity analysis (lecanemab vs. aducanumab). The horizontal axis represents the incremental cost-effectiveness ratio (ICER, $/QALY gained). MCI, mild cognitive impairment; SoC, standard of care; HR, hazard ratio.

According to the results of the OSA, the HR of MCI for mild dementia due to AD between aducanumab and SoC, the utility of MCI, and the cost of aducanumab were the three most sensitive parameters to the ICER of the aducanumab group compared to the SoC group (Supplementary Figure 1). The top factors that impacted the ICER of the lecanemab group compared to the SoC group were the initial age of the SoC group and the initial age of the lecanemab group (Supplementary Figure 2). For the ICER of the lecanemab group compared to the aducanumab group, the utility of the state of mild dementia due to AD, the utility of the state of MCI, and the aducanumab or lecanemab cost had the most important effects (Figure 2).

**Figure 3:**
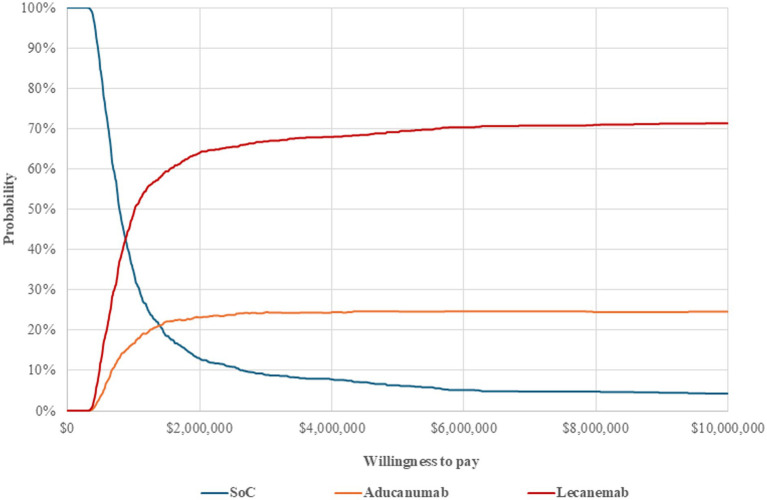
Cost-effectiveness acceptability curves (CEACs). SoC, standard of care.

The results of probabilistic sensitivity analysis showed that lecanemab was more cost-effective than aducanumab across various WTP thresholds. When the WTP was over $880,000, there was a higher likelihood that lecanemab was more cost-effective than SoC (Figure 3). For aducanumab, the WTP should reach $1,400,000.

## Discussion

Lecanemab’s launch price is $26,500 per patient per year (23), and it was found to not be cost-effective when the WTP is $150,000. The lecanemab group (vs. the SoC group) had an ICER of $409,488.34 per QALY gained. Aducanumab had an ICER of $1,354,544.47 per QALY gained when compared to the SoC group. Both one-way sensitivity analysis and probabilistic sensitivity analysis showed that these results were robust. When indirect comparisons of the cost-effectiveness of these drugs were used, we found that lecanemab had an ICER of $121,678.49 per QALY gained when compared with aducanumab, suggesting that lecanemab may be more effective than aducanumab.

The results of one previous cost-effectiveness analysis documented that aducanumab created minimal health improvements compared to its cost, with the ICER of $1.33 million per QALY gained from the health care system perspective (14). It was also determined that for aducanumab to meet cost-effectiveness thresholds, it would have to be sold at an 85–95% discount from the annual launch price (14). Another study with a 5-year time horizon indicated that aducanumab would be cost-effective at $22,820 per year, which is lower than its current price even after the reduction (15). Hence, aducanumab was deemed not cost-effective, and the annual price was deemed unreasonable, which is consistent with our study.

An earlier microsimulation study using only phase II clinical trials compared lecanemab with the SoC vs. the SoC alone and concluded that lecanemab would have a potential annual value-based price (VBP) of $26,820 from a payer perspective based on a WTP threshold of $150,000 per QALY gained (31), indicating that lecanemab would be cost-effective. However, the most recent study on the cost-effectiveness of lecanemab stated that lecanemab treatment, whether targeted or unrestricted by APOE ε4 genotype, is not cost-effective compared to SoC alone for patients with MCI or mild dementia due to AD (16). Lecanemab could be cost-effective in certain scenarios if priced below $5,100 per year, which is much lower than the current price (16).

Neither aducanumab nor lecanemab would be cost-effective compared to SoC at their current prices; however, our study indicated that lecanemab, when compared with aducanumab, would be cost-effective. Additionally, it is estimated that when lecanemab is covered by a patient’s insurance, approximately 91% of individuals will have coverage through Medicare with Medigap, Medicare Advantage, Medicaid, or commercial insurance, which will lead to an out-of-pocket cost of $0 to a few dollars per day (23). The remaining individuals, who have Medicare without supplemental insurance, will be responsible for 20% of lecanemab’s cost as co-insurance under Medicare Part B, with an out-of-pocket expense of approximately $14.50 per day (23). For those who qualify, such as individuals without insurance or those on Medicare who meet financial or other program criteria, Eisai is establishing a Patient Assistance Program to provide lecanemab at no cost (23).

On 6 July 2023, the FDA granted full approval to lecanemab (8). As a result, Medicare will provide extensive coverage for this medication. It was estimated that approximately 100,000 people would use lecanemab by the third year, potentially leading to $2.7 billion in annual Medicare Part B spending (32). If adoption increases to 5% or 10% of AD patients, costs could rise to $8.9 billion or $17.8 billion annually (32). The Inflation Reduction Act (IRA) aims to address high drug costs by allowing Medicare to negotiate prices for top-spending drugs. However, manufacturers of biologics such as lecanemab are exempt from these negotiations for 13 years following the drug’s approval. This means lecanemab’s manufacturers have until 2036 to recover their R&D investments and generate revenue before negotiated prices might apply (32). While lecanemab may offer some clinical benefits to older adults with early-stage AD, it could significantly increase Medicare spending and premiums, posing future challenges for Medicare, patients, and taxpayers.

Additionally, extra caution is necessary when administering lecanemab, particularly because it will commonly be used in older patients. Lecanemab may lead to adverse events such as ARIA and infusion reactions. The Appropriate Use Recommendations (AURs) (33) advise against prescribing lecanemab to patients on anticoagulants due to a heightened risk of hemorrhage. Furthermore, patients carrying the APOE ε4 gene, especially homozygotes, are at an increased risk of ARIA, and the AURs (33) suggest APOE genotyping to guide risk discussions. Clinician and institutional preparedness are crucial, with protocols needed to manage serious events. Clear communication between clinicians and patients or their caregivers is vital to ensure they fully understand the potential benefits, risks, and monitoring requirements associated with lecanemab treatment.

Although aducanumab is no longer marketed, this comparison remains important. Aducanumab was the first FDA-approved *β*-amyloid-targeting therapy, and lecanemab was the second, making it essential to contextualize lecanemab’s value relative to its predecessor and to understand how improvements in efficacy, safety, and cost shape overall cost-effectiveness. This analysis also provides a framework for comparative economic evaluation of anti-amyloid therapies, supporting pricing, reimbursement, and negotiation decisions for stakeholders. In addition, it offers a reference case illustrating lecanemab’s economic value had aducanumab remained available. No published studies have directly compared the cost-effectiveness of these agents, and the continued academic engagement with aducanumab in the literature underscores the relevance of such a comparison. Without this analysis, decision-makers would lack the critical comparative information needed to guide formulary decisions, treatment pathways, and resource allocation.

Indirect comparisons are commonly used and methodologically accepted in pharmacoeconomic evaluations; however, differences in baseline characteristics, trial design, and endpoint implementation between the lecanemab and aducanumab trials (6, 10) may bias the HRs informing the comparison. Specifically, the lecanemab trial enrolled more participants with mild dementia, greater racial diversity, and slightly higher baseline CDR-SB scores, which is associated with faster progression and wider apparent treatment–placebo separation. Trial-level differences, including the evolving APOE ε4-dependent titration and ARIA-related dose interruptions in the aducanumab trials vs. fixed dosing in the lecanemab trial, may further influence treatment exposure and observed effects. Endpoints are consistent across the trials, but variations in endpoint timing and operational procedures can likewise affect measured progression rates. Together, these sources of heterogeneity may shift HRs in either direction and therefore represent an important source of uncertainty in the indirect comparison. To address these concerns, we conducted extensive one-way and probabilistic sensitivity analyses to evaluate how alternative plausible HRs would influence the cost-effectiveness results.

This study has several important strengths. It is the first to directly compare the cost-effectiveness of lecanemab and aducanumab, thereby addressing a significant gap in the existing literature. By incorporating real-world Medicare data to estimate health service costs, the analysis reflects the actual expenditures incurred in clinical practice, enhancing the validity and applicability of the findings. Furthermore, the study improves the precision of its estimates by calculating transition probabilities across specific age groups, enabling a more accurate evaluation of the economic impact of these therapies on different patient populations. Taken together, these features strengthen the robustness and relevance of the analysis for informing clinical, policy, and payer decision-making.

This study has several limitations. First, because no head-to-head clinical trials comparing lecanemab and aducanumab are available, we relied on an indirect comparison, which introduces uncertainty in estimating their relative effectiveness. Nevertheless, extensive one-way and probabilistic sensitivity analyses demonstrated that our findings were robust to this uncertainty. Second, differences in baseline characteristics and trial designs, including a higher proportion of participants with mild dementia and greater racial diversity in the lecanemab trial, may influence disease progression rates and affect the comparability of HRs, potentially biasing the indirect comparison. Third, ARIA rates were based on clinical trial data because real-world evidence remains limited, which may not fully reflect the true incidence of ARIA in clinical practice, particularly among higher-risk subgroups such as APOE ε4 carriers. Future studies based on high-quality real-world data are urgently needed. In addition, our model captured only ARIA-related adverse events and did not include other serious events such as infusion reactions, hypersensitivity reactions, or treatment-related discontinuations, which may lead to underestimation of total adverse event-related costs and overall resource use. The ARIA-related cost estimates were based on the assumption that each event required three MRI scans, although actual resource use may be higher in certain patients. Furthermore, because this analysis was conducted from the perspective of the healthcare system, broader societal costs, which include caregiver burden and productivity losses, were not incorporated, which may underestimate the overall economic impact of AD. Finally, our model did not include post-trial reports of deaths associated with lecanemab (34) because clinical trials are not designed to estimate long-term mortality risks, and reliable incidence estimates are not yet available. As real-world pharmacovigilance data become available, future cost-effectiveness analyses should incorporate these additional mortality risks to provide a more comprehensive assessment of long-term safety and economic value.

## Conclusion

Our findings indicate that lecanemab is more cost-effective than aducanumab; however, at current prices, neither therapy is cost-effective compared with SoC for early AD in the US. Price reduction for lecanemab, which is covered by Medicare, is warranted to achieve favorable cost-effectiveness. Yet under the current IRA, biologics such as lecanemab are exempt from Medicare price negotiations for 13 years after approval, limiting near-term opportunities for cost adjustment.

## Data Availability

The data that support the findings of this study are available from the corresponding author upon reasonable request.
