# Supplementary material for: Lifetime cost-effectiveness of lecanemab for early Alzheimer’s disease
Source: Front Public Health. 2026 Jan 30;14:1692508. doi: 10.3389/fpubh.2026.1692508 (PMC12901472; doi:10.3389/fpubh.2026.1692508)
Supplement: Supplementary file 1 [file Supplementary_file_1.docx]

| Supplementary Table 1. Sensitivity analysis results with different utility values | | | | | |  |
| --- | --- | --- | --- | --- | --- | --- |
|  | Incremental costs, US$ | Incremental LYs | Incremental QALYs | ICER, US$/LY | ICER, US$/QALY |  |
| Aducanumab vs. SoC | 101,770.83 | 0.08 | 0.08 | 1,237,130.61 | 1,206,985.25 |  |
| Lecanemab vs. SoC | 131,789.80 | 0.33 | 0.35 | 395,747.84 | 372,125.10 |  |
| Lecanemab vs. Aducanumab | 30,018.97 | 0.25 | 0.27 | 119,716.27 | 111,248.80 |  |
| Utility values for different health states are as follows: 0.80 for mild cognitive decline (MCI), 0.74 for mild dementia, 0.59 for moderate dementia, and 0.36 for severe dementia. | | | | | |  |
|  |  |  |  |  |  |  |
| LY: Life year; QALY: Quality-adjusted life year; ICER: Incremental cost-effectiveness ratio; SoC: standard of care | | | | | |  |

| Supplementary Table 2. Sensitivity analysis results with different hazard ratios for disease progression | | | | | | |
| --- | --- | --- | --- | --- | --- | --- |
|  |  | Incremental costs, US$ | Incremental LYs | Incremental QALYs | ICER, US$/LY | ICER, US$/QALY |
| HR = 0.8 | |  |  |  |  |  |
|  | Aducanumab vs. SoC | 136,928.50 | 0.93 | 0.61 | 147,086.73 | 225,225.93 |
|  | Lecanemab vs. SoC | 159,127.97 | 1.10 | 0.77 | 145,313.29 | 206,285.75 |
|  | Lecanemab vs. Aducanumab | 22,199.47 | 0.16 | 0.16 | 135,254.53 | 135,830.39 |
| HR = 1.2 | |  |  |  |  |  |
|  | Aducanumab vs. SoC | 76,331.48 | -0.54 | -0.31 | -141,283.92 | -243,957.77 |
|  | Lecanemab vs. SoC | 110,455.87 | -0.24 | -0.02 | -458,258.70 | -5,169,324.62 |
|  | Lecanemab vs. Aducanumab | 34,124.39 | 0.30 | 0.29 | 114,038.27 | 117,056.55 |
| LY: Life year; QALY: Quality-adjusted life year; ICER: Incremental cost-effectiveness ratio; HR: hazard ratio; SoC: standard of care | | | | | | |

Supplementary Fig. 1. Tornado diagram of the result of one-way sensitivity analysis (aducanumab vs. SoC)
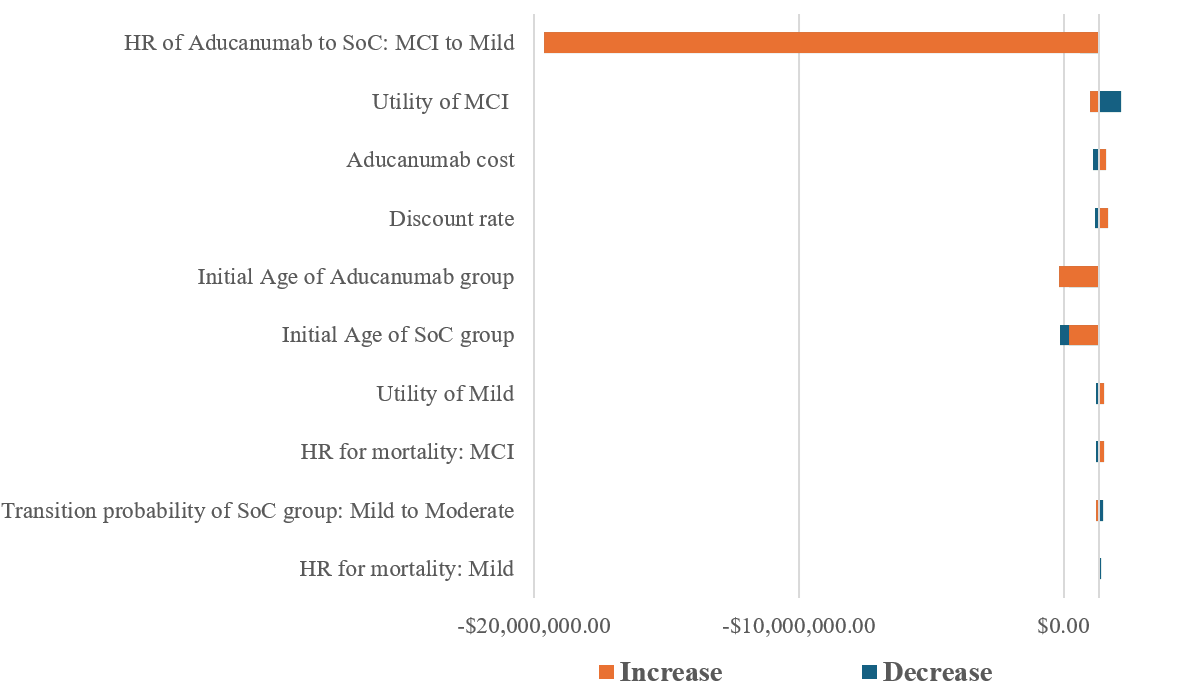
MCI: mild cognitive impairment; SoC: standard of care; HR: hazard ratio.

Supplementary Fig. 2. Tornado diagram of the result of one-way sensitivity analysis (lecanemab vs. SoC)


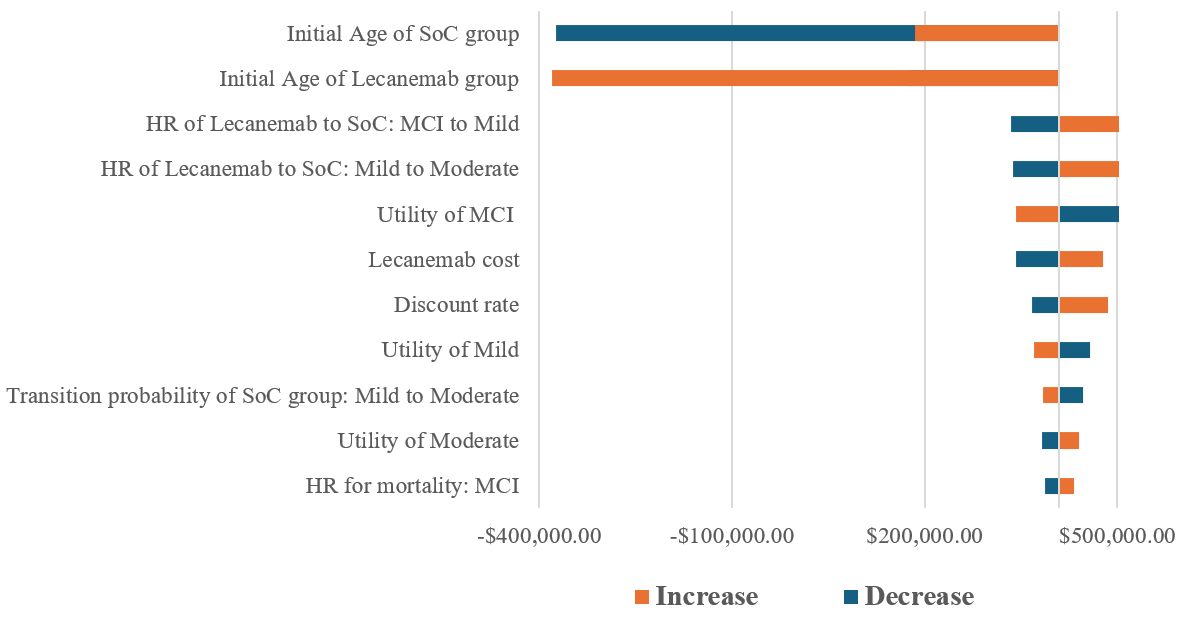
MCI: mild cognitive impairment; SoC: standard of care; HR: hazard ratio.
